# Regulation of Pleiotrophin and PTPRZ1 Expression by Hypoxia to Restrict Hypoxia-Induced Cell Migration

**DOI:** 10.3390/cancers17091516

**Published:** 2025-04-30

**Authors:** Evangelia Poimenidi, Eirini Droggiti, Katerina Karavasili, Dimitra Kotsirilou, Eleni Mourkogianni, Pieter Koolwijk, Evangelia Papadimitriou

**Affiliations:** 1 Laboratory of Molecular Pharmacology, Department of Pharmacy, University of Patras, 26504 Patras, Greece; evipoi@yahoo.com (E.P.);; 2 Department of Physiology, Amsterdam UMC, 1081 HV Amsterdam, The Netherlands

**Keywords:** angiogenesis, cancer, endothelial cells, hypoxia, integrin, tyrosine phosphatase

## Abstract

Background/Objectives: In the tumor microenvironment, hypoxia regulates genes that support tumor cell invasion and angiogenesis under the control of the hypoxia-inducible transcription factors (HIFs). Pleiotrophin (PTN) is a secreted protein that activates cell migration in endothelial and cancer cells that express α_ν_β_3_ integrin but has inhibitory effects in cells that do not express α_ν_β_3_ integrin. In both cases, the protein tyrosine phosphatase receptor zeta 1 (PTPRZ1) seems to mediate the effects of PTN. In the present work, we studied the effect of hypoxia on PTN and PTPRZ1 expression and the functional consequences of this effect. Methods: Western blot, quantitative real-time PCR, and luciferase assays were used to study the impact of hypoxia at the protein, mRNA, and transcriptional levels, respectively. Decoy oligonucleotides (ODNs), siRNA technology, and plasmid overexpression were used to study the involvement of the transcription factors studied. Functional assays were used to study the effect of hypoxia on cell proliferation and migration. Results: Hypoxia increases PTN expression through the transcriptional activation of the corresponding gene in α_ν_β_3_ integrin-expressing cells. The transcription factors HIF-1α, HIF-2α, and AP-1 mediate the up-regulation of PTN by hypoxia. Functional assays in endothelial cells from PTN knockout mice or endothelial and cancer cells following the downregulation of PTN expression showed that PTN negatively affects chemical hypoxia-induced cell proliferation and migration. In cancer cells that do not express α_ν_β_3_ integrin, hypoxia or chemical hypoxia inhibits PTN expression in a HIF-1α-, HIF-2α-, and AP-1-independent manner. The expression of PTPRZ1 is up-regulated by chemical hypoxia, is HIF-1α- and HIF-2α-dependent, and seems to limit the activation of HIF-1α, at least in endothelial cells. Conclusions: Hypoxia or chemical hypoxia regulates PTN and PTPRZ1 expressions to restrict the stimulatory effects of hypoxia on endothelial and cancer cell migration.

## Simple Summary

Hypoxia regulates gene expression to support tumor cell invasion and angiogenesis. Hypoxia-inducible transcription factors (HIFs) play a major role in the hypoxia effects through interactions with other transcription factors, such as AP-1. Pleiotrophin (PTN) acts through protein tyrosine phosphatase receptor zeta 1 (PTPRZ1) and regulates cell migration in a manner that depends on α_ν_β_3_ integrin expression. The present study shows that hypoxia or chemical hypoxia increases the PTN expression in endothelial and glioblastoma cells that express α_ν_β_3_ integrin in a HIF- and AP-1-dependent manner to negatively impact the stimulatory effect of hypoxia on endothelial cell proliferation and migration. The expression of PTPRZ1 is also enhanced by hypoxia, is HIF-dependent, and limits the activation of HIF-1α in endothelial cells. In conclusion, hypoxia or chemical hypoxia regulates PTN and PTPRZ1 expression to restrict its stimulatory effects on endothelial and cancer cells.

## 1. Introduction

Hypoxia is present in several physiological and pathological conditions due to a decreased supply or increased demand for O_2_, and all organisms have developed mechanisms to adapt to hypoxia. The key mediators of this adaptive response are the hypoxia-inducible factors (HIFs) that trigger the transcription of multiple genes and regulate processes, such as glucose metabolism, cell survival, proliferation, apoptosis, and angiogenesis. To date, three isoforms of HIFs have been found: HIF-1, HIF-2, and HIF-3. HIFs are heterodimers comprising a constitutively expressed beta (β) subunit and a regulated alpha (α) subunit. In the presence of oxygen, the α subunit is hydroxylated on two prolines by specific oxygen prolyl hydroxylases, and the hydroxylated form interacts with the von Hippel–Lindau protein, which targets this subunit for degradation by the proteasome. Under hypoxic conditions, the α subunit is not hydroxylated, which results in its stabilization and translocation into the nucleus, where it dimerizes with the β subunit, forming the active HIF transcription factor that binds to the hypoxia response element (HRE) present in the promoter of its target genes [1,2]. Another redox-sensitive, hypoxia-activated transcription factor is AP-1, which plays roles in cell proliferation, apoptosis, inflammation, migration, differentiation, and angiogenesis by affecting the expression of numerous genes [3,4,5,6], including pleiotrophin [7].

Pleiotrophin (PTN) is a secreted growth factor that induces migration in cells expressing α_ν_β_3_ integrin, including endothelial cells [8,9]. However, PTN has also been shown to inhibit cell migration in cells that lack α_ν_β_3_ integrin expression [9,10]. Among the numerous receptors that regulate the effects of PTN on endothelial cells [11], the protein tyrosine phosphatase receptor zeta 1 (PTPRZ1) is of great importance [8,12]. PTPRZ1 plays a key role in regulating cell migration [13] and acts as a receptor for cytokines and growth factors, e.g., VEGFA_165_ [12,14]. PTN is upregulated by hypoxia in rat hepatic stellate cells [15], in a rat model of myocardial infarction [16], or after acute ischaemic brain injury [17]. It remains unclear, however, how hypoxia affects PTN expression and whether PTN has a role in the effects of hypoxia in different types of cells. On the other hand, PTPRZ1 is up-regulated by both the HIF-2α [18,19] and HIF-1α [20] that act through the HREs found in the *Ptprz1* gene promoter. The functional significance of the PTPRZ1 up-regulation by hypoxia is still unknown.

In the present work, we investigated the effect of hypoxia on the expression of PTN and PTPRZ1, identified the role of the HIFs and AP-1 transcription factors, and clarified their functional significance on hypoxia-induced cell migration.

## 2. Materials and Methods

### 2.1. Cells

The endothelial cells used in the present study were human umbilical vein endothelial cells (HUVECs), and lung microvascular endothelial cells (LMVECs) derived from wild-type C57BL/6J mice (*Ptn*^+/+^), C57BL/6J knockout mice for PTN (*Ptn*^−/−^), wild-type SV129/B6 mice (*Ptprz1^+/+^*), and SV129/B6 knockout mice for PTPRZ1 (*Ptprz1^−/−^*). In all cases, endothelial cells were isolated as previously described [12,21]. We also used human glioblastoma U87MG and M059K cells, rat C6 glioma cells [9], and human prostate cancer LNCaP cells that were stably transfected with the pCDNA3.1 control vector (LNCaP-PC), or with the same vector carrying an antisense *ptn* gene (LNCaP-AS) [22]. Cell culture media contained penicillin 100 u/mL /streptomycin 100 μg/mL, gentamycin 50 μg/mL, and amphotericin 2.5 μg/mL, and cultures were maintained at 37 °C, 5% CO_2_, and 100% humidity.

### 2.2. Hypoxia

A custom-designed hypoxic workstation (T.C.P.S, Rotselaar, Belgium) equipped with an oxygen transmitter (GE Panametrics, Billerica, MA, USA), which includes a CO_2_- and O_2_-controlled humidified incubator (Sanyo, Ettenleur, The Netherlands) placed inside a T4 glove box (Jakomex, Dagneux, France), was used. An internal zirconia sensor monitored the oxygen concentration. All cell culture media and buffers were pre-incubated into the hypoxic workstation for 4 h before use [23]. The Anaerocult^®^ A system (Merck Millipore, Darmstadt, Germany, #1.01611.0001) created the hypoxic 0.2% O_2_ atmosphere. Chemical hypoxia was achieved by incubating cells with either deferoxamine (DFX) at a final concentration of 200 μM (deferoxamine mesylate, Merck, Germany, #252750) or dimethyloxalylglycine (DMOG), at a final concentration of 0.5 mM (Santa Cruz Biotechnology, Heidelberg, Germany, #sc-200755). These concentrations induced optimal up-regulation of HIF-1α (Supplementary Figure S1A). DFX chelates iron leading to the accumulation of HIF-α and its translocation from the cytoplasm to the nucleus, where it dimerizes with HIF-1β, to become transcriptionally active [24]. DMOG is a cell-permeable, competitive inhibitor of the prolyl-hydroxylases that stabilize HIF-α [1]. When cells reached 80–90% confluence, they were serum-starved with medium containing 5% FBS for 16 h before treatment with DFX, DMOG, or low O_2_. The effect of the different types of hypoxia on the levels of PTN expression was not the focus of this study since the Western blot assay has the limitation of being semi-quantitative.

### 2.3. Transient Cell Transfection

Plasmid pEGFP-HIF-1α and pEGFP-HIF-2α constructs have been previously described [25,26]. The pcDNA3.1-GFP vector was used as a control. C6 and M059K cells, at 50% confluency, were incubated with the plasmid DNA and the suitable transfection reagents, which were jetPEI™ in C6 cells or jetPEI-HUVEC™ in M059K cells (both from Polyplus Transfection, #101-10N and #108-01N, respectively). In both cases, a N/p = 5 ratio was used, and cells were incubated with the transfection medium for 4 h at 37 °C and then with fresh serum-containing medium 24 h later before assaying for the expression of HIFs and VEGFA (positive control), PTN, or PTPRZ1 by Western blot analysis.

### 2.4. RNA Interference

To down-regulate PTPRZ1 expression, C6 cells or LNCaP cells at 60% confluency were incubated in a transfection medium (Santa Cruz Biotechnology Inc., #sc-36868) containing 80 nM annealed siRNA for PTPRZ1 [14,27] and siRNA transfection reagent (Santa Cruz Biotechnology Inc., #sc-29528) for 6 h. The medium was replaced with the corresponding full medium, and cells were cultured for 48 h before being used for further experiments. To down-regulate HIF-1α or HIF-2α, HUVECs (30 × 10^4^ on 10 cm^2^) were transfected in 2 mL culture medium containing 2.5 μL DharmaFECT transfection reagent Type 1 (GE Dharmacon Lafayette, Lafayette, CO, USA) and 1 μg siRNA (dssiRNA for HIF-1α or HIF-2α, Qiagen, Venlo, The Netherlands). HUVECs were placed in the electroporation system (Amaxa, Lonza Verviers, Belgium) according to the manufacturer’s instructions, and cultured for 18 h after transfection, before starting further experiments [23]. Double-stranded negative control siRNA (Ambion Inc., Austin, TX, USA, Silencer^R^ Negative Control siRNA, #AM4635) was used in all cases.

### 2.5. Western Blot Analysis

Proteins were analyzed by SDS-PAGE and transferred to Immobilon P membranes. Blocking was performed by incubating the membranes with Tris-buffered saline (TBS), pH 7.4, with 0.1% Tween, containing 5% nonfat dry milk. Membranes were further incubated with PTN monoclonal antibody (M01) clone 5C3 (1:1000, Abnova, Heidelberg, Germany, #H00005764-M01), mouse anti-PTPRZ1 (1:500, BD Biosciences (Franklin Lakes, NJ, USA, #610180), GFP (D5.1) rabbit mAb (1:200, Cell Signaling Technology (Danvers, MA, USA, #2956), rabbit polyclonal VEGF Antibody (A-20) (1:250, Santa Cruz Biotechnology, #sc-152), HIF-1α (D1S7W) XP^®^ Rabbit mAb (1:1000, Cell Signaling Technology, #36169), or mouse monoclonal actin antibody (2Q1055) (1:2000, Santa Cruz Biotechnology, #sc-58673) for 16 h at 4 °C under continuous agitation, washed 3 times with TBS-T, and incubated with horseradish peroxidase-conjugated anti-mouse IgG (Cell Signaling Technology; #7076) or anti-rabbit IgG (Cell Signaling Technology; #7074) for 1 h at room temperature. The membranes were washed, and the immunoreactive bands were detected using the enhanced chemiluminescence (ECL) detection kit (Pierce Biotechnology, Rockford, IL, USA), according to the manufacturer’s instructions. The protein levels corresponding to the immunoreactive bands were quantified using the ImageJ image analysis software (version 1.53e). Unless otherwise stated, the PTN/PTPRZ1 signal was normalized to the β-actin (loading control) signal for the corresponding sample.

### 2.6. Quantitative Real-Time PCR Analysis

RNA isolation of HUVECs from at least 4 donors was performed using the RNeasy Mini Kit (Qiagen, Venlo, The Netherlands). cDNA was synthesized using the Cloned AMV First Strand c DNA Synthesis Kit from Invitrogen (Waltham, MA, USA) with poly(T)primers. Oligonucleotides were designed and used as previously described [28]. Quantitative real-time PCR using SYBR Green was performed in duplicate wells in an ABI 7500 sequence detection system (Applied Biosystems, Foster City, CA, USA). As an endogenous reference gene, we used β2-microglobulin, and the relative expression level of PTN was calculated using the comparative Ct method, as previously described [28].

### 2.7. Transient Transfection and Luciferase Assay

Plasmid constructs containing the full-length promoter of the wild-type human *ptn* gene or the full-length promoter of the human *ptn* gene carrying point mutations in both AP-1-like motifs, fused to a luciferase reporter gene, were used as previously described [7]. U87MG or M059K cells were cultured in 12-well plates (4 × 10^5^ cells/well) overnight, before being incubated in 1 mL of serum-free medium containing 1.5 μg of plasmid DNA and 4 μL of jetPEI™ transfection reagent, for 4 h at 37 °C. The transfection medium was replaced by a fresh medium, and 16 h later, the culture medium was replaced with a medium containing DFX for different periods. Luciferase activity was determined using the luciferase reporter gene assay from Roche Applied Science (Indianapolis, IN, USA), according to the manufacturer’s instructions. Cell lysates were analyzed for protein content using the Bradford method, and luminescence units were normalized for total protein content [7].

### 2.8. Decoy Oligonucleotide (ODN) Technique

The single-stranded phosphorothioate-bonded ODNs for AP-1 and HIFs (HIF-1 and HIF-2) used in the present study are based on previous studies [7,29] and shown in Supplementary Table S1. Complementary single-stranded ODNs were incubated at 95 °C for 5 min followed by a cool-down phase of 3 h at ambient temperature, to form the double-stranded ODNs to be used. The efficiency of the hybridization reaction was verified in 2.5% agarose gels and always exceeded 95%. HUVECs were seeded in 6-well plates at 80% confluency and after 24 h were incubated with the double-stranded ODNs (450 nM) and 6 μL of jetPEI™ in 1 mL of serum-free medium for 4 h before performing further experiments.

### 2.9. Proliferation Assay

LMVECs were plated in 48-well plates (1.5 × 10^4^ cells/well). When cells reached 70–80% confluency, they were serum-starved overnight and then incubated with the tested agents for 24 h in the serum-starved medium. The cells were then harvested by trypsin and counted using a hemocytometer.

### 2.10. Migration Assays

Migration assays were performed in 24-well microchemotaxis chambers (Corning, Inc., Lowell, MA, USA) using uncoated polycarbonate membranes with 8 μm pores. Cells were serum-starved and suspended in serum-free medium containing 0.25% BSA (10^5^ cells/0.1 mL). The bottom chamber was filled with 0.6 mL of serum-free medium containing 0.25% BSA and the tested agents whenever applied. The upper chamber was loaded with 0.1 mL of the cell suspension and the transwell was incubated for 4 h at 37 °C. The cells on the membranes were fixed, stained with 0.33% toluidine blue or 1% crystal violet solution, and, in the case of endothelial cells, quantified on the entire area of each membrane using a grid and a microscope (Optech Microscope Services Ltd., Oxfordshire, UK) using the 20Χ objective. Two independent researchers blindly counted each membrane. In the case of cancer cells, crystal violet was extracted from the membranes by 200 μL of a 10% acetic acid solution, transferred to 96-well plates, and counted on a photometer at 630 nm.

### 2.11. Statistical Analysis

All data is derived from at least three independent experiments. All results are expressed as mean ± SD, and the significance of variability between the experimental and the corresponding control groups was determined by unpaired *t*-test or ANOVA as required.

## 3. Results

### 3.1. Hypoxia Stimulates PTN Expression Through Increased Transcription of Corresponding Gene

**Figure 1:**
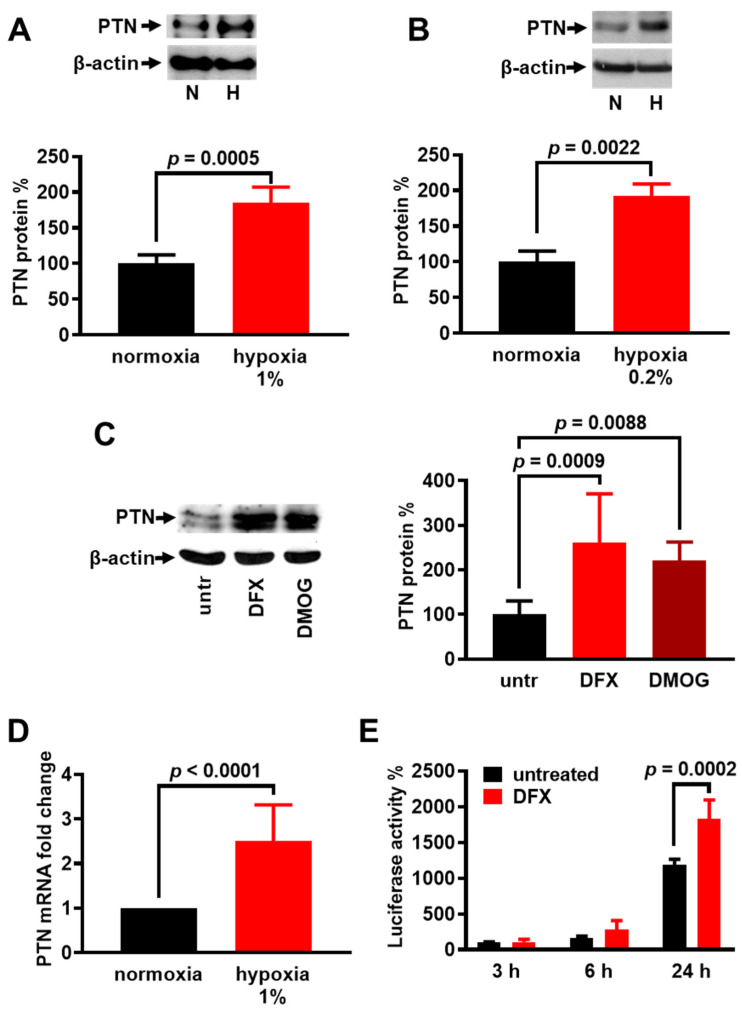
Hypoxia enhances the expression of PTN in endothelial cells. (**A**–**C**) Representative Western blot for PTN and β-actin following a 24 h incubation of HUVECs in an oxygen atmosphere of 1% (**A**), in Anaerocult A bags (hypoxia 0.2%) (**B**), or in a treatment with DFX (200 μM) or DMOG (0.5 mM) (**C**). Results are expressed as the mean ± SD (n = 3) of the percentage change in the PTN protein levels compared with the corresponding normoxia or untreated cells (untr, set by default as 100%). N, normoxia; H, hypoxia. (**D**) PTN mRNA levels after HUVEC incubation in an oxygen atmosphere of 1% for 24 h. Results are expressed as the mean ± SD (n = 3) of the percent change in the PTN mRNA level compared to the normoxia-treated cells. (**E**) U87MG cells were transfected with the full-length promoter of the wild-type human *AP-1* gene fused to a luciferase reporter gene. The luciferase activity at different time points after adding DFX in the cell culture medium was normalized to the total protein content. Results are expressed as the mean ± SD (n = 3) of the percent change in the luciferase activity compared to the untreated cells (untr) at 3 h (set by default as 100%).

To study whether hypoxia affects the PTN expression by HUVECs, we cultured the cells under hypoxic conditions and measured PTN protein amounts normalized to the corresponding β-actin protein amounts. As shown in Figure 1, PTN protein levels in HUVECs were significantly increased 24 h after the application of hypoxia, both in a hypoxic station (Figure 1A) and in Anaerocult A bags (Figure 1B), as well by the application of chemical hypoxia by incubating HUVECs with DFX or DMOG (Figure 1C). PTN mRNA levels were significantly increased 24 h after hypoxia application in HUVECs (Figure 1D), suggesting that hypoxia enhances PTN expression. To evaluate whether the effect of hypoxia on PTN is observed at the transcriptional level, we used a plasmid construct containing the full-length promoter of the human PTN gene fused to a luciferase reporter gene [7] to transfect U87MG cells. We could not use HUVECs for such assays, due to their inefficient transfection with the reporter gene. U87MG cells were chosen because they express α_ν_β_3_ integrin and PTN enhances their migration, similarly to HUVECs [9]. Before performing the luciferase assays, we verified that chemical hypoxia up-regulates PTN protein levels in U87MG cells (Supplementary Figure S1B). The reporter activity was increased in response to chemical hypoxia (Figure 1E), suggesting that hypoxia up-regulates PTN expression at the transcriptional level.

### 3.2. HIF-1α and HIF-2α Are Involved in the Hypoxia-Induced PTN Up-Regulation

**Figure 2:**
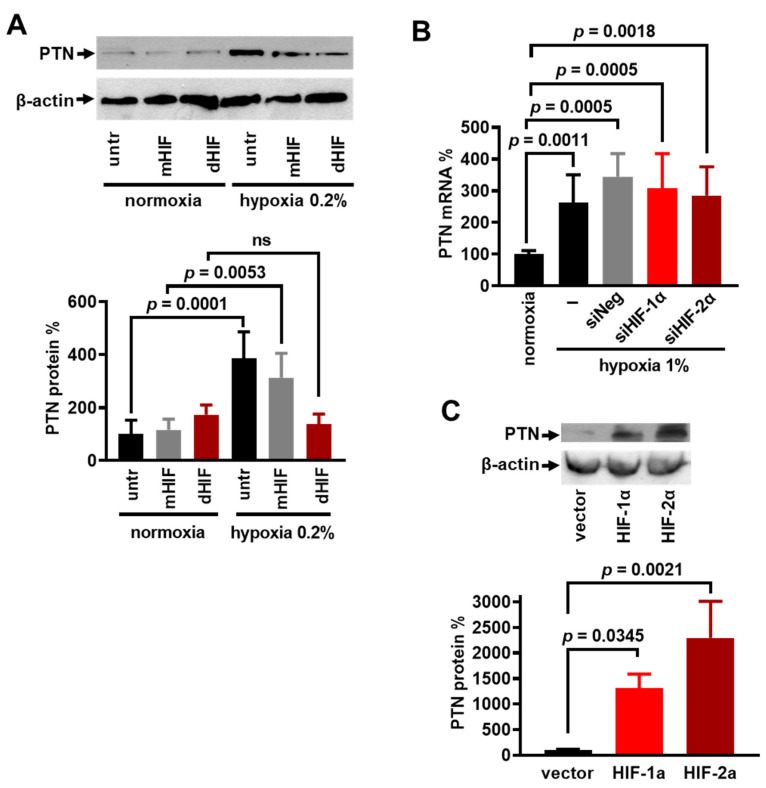
HIFs are involved in the hypoxia-induced PTN up-regulation in endothelial cells. (**A**) The representative Western blot for PTN following a 24 h incubation of HUVECs in 0.2% oxygen. Cells were either untreated (untr) or treated with ODNs containing the HIF-α consensus sequence (dHIF) or treated with ODNs containing the mutated HIF-α consensus sequence (mHIF). Results are expressed as the mean ± SD (n = 4) of the percent change in the relative PTN protein levels compared with the untreated cells cultured in normoxic conditions. ns, non-significant. (**B**) PTN mRNA levels after HUVEC incubation in 1% oxygen for 24 h following the downregulation of HIF-1α (siHIF-1α) or HIF-2α (siHIF-2α). siNeg corresponds to cells treated with a negative control siRNA sequence. Results are expressed as the mean ± SD (n ≥ 3) of the percent change in PTN mRNA levels compared to the corresponding normoxia-treated cells (set by default as 100% in each case). (**C**) U87MG cells were transfected with pEGFP-HIF-1α (HIF-1α), pEGFP-HIF-2α (HIF-2α), or the pcDNA3.1-GFP vector (vector). Cell lysates were analyzed by a Western blot for PTN and β-actin, and results are expressed as the mean ± SD (n = 3) of the percentage change in the PTN protein levels compared with the untreated cells.

To determine whether HIFs are involved in the hypoxia-induced PTN expression, we used HIF ODNs (dHIF) that inhibit both HIF-1α and HIF-2α [29]. When HUVECs were transfected with ODNs containing a mutated HRE consensus sequence (mHIF), hypoxia upregulated the PTN expression, like its effect on untransfected HUVECs. At the same time, the transfection with dHIF ODNs inhibited the stimulatory effect of hypoxia (Figure 2A). To see whether this effect is specifically due to HIF-1α or HIF-2α, we down-regulated their expression by siRNA in HUVECs (Supplementary Figure S2A), as previously described [23]. The decreased HIF-1α or HIF-2α expression did not affect the hypoxia-induced mRNA levels of PTN (Figure 2B). On the other hand, the overexpression of HIF-1α or HIF-2α in U87MG cells (Supplementary Figure S2B) increased the PTN protein levels (Figure 2C). Altogether, these data support a redundant role of HIFs in the PTN up-regulation by hypoxia.

### 3.3. AP-1 Is Involved in the Hypoxia-Induced PTN Expression

**Figure 3:**
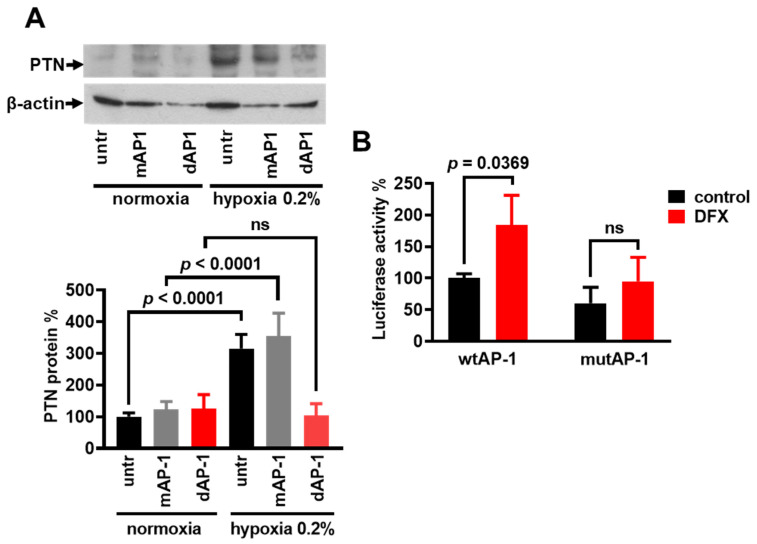
AP-1 is involved in the hypoxia-induced PTN expression. (**A**) The representative Western blot for PTN following a 24 h incubation of HUVECs in Anaerocult A bags (hypoxia 0.2%). Cells were either untreated (untr) or treated with ODNs containing the AP-1 consensus sequence (dAP-1) or treated with ODNs containing the mutated AP-1 consensus sequence (mAP-1). Results are expressed as the mean ± SD (n = 4) of the percentage change in the PTN protein levels compared with the untreated cells cultured in normoxic conditions. (**B**) U87MG cells were transfected with the full-length promoter of the wild-type human *AP-1* gene (wtAP-1) or the full-length promoter of the human *ptn* gene carrying point mutations in both AP-1-like motifs (mutAP-1), fused to a luciferase reporter gene. The luciferase activity 24 h after adding DFX (200 μM) in the cell culture medium was normalized to the total protein content. Results are expressed as the mean ± SD (n = 3) of the percent change in the luciferase activity compared to the untreated cells transfected with the wtAP-1 construct (set by default as 100%). ns, non-significant.

Since hypoxia is also known to activate the AP-1 transcription factor [3,4,5,6], and the *ptn* gene promoter contains two AP-1 binding sites [7], we tested whether the hypoxia-induced PTN expression is due to hypoxia-induced AP-1 activation. The transfection of HUVECs with AP-1 ODNs before the hypoxia application resulted in marked attenuation of the hypoxia-induced PTN protein up-regulation. ODNs containing a mutated AP-1 consensus sequence had no effect (Figure 3A). We then examined the contribution of the AP-1 motifs of the human *ptn* promoter to the increased transcription of the *ptn* gene in hypoxia-treated U87MG cells. For this reason, we used the plasmid construct containing the full-length promoter of the human *ptn* gene fused to a luciferase reporter gene, as well as a construct with point mutations in both AP-1-like motifs [7] to transfect U87MG cells, as described in the Materials and Methods Section. Chemical hypoxia increased the reporter activity of the wild-type *ptn* promoter (wtAP-1) but not the reporter activity of the AP-1 double mutant (mutAP-1), suggesting that AP-1 may be involved in the hypoxia-induced transcription of the *ptn* gene (Figure 3B).

### 3.4. PTN Expression Inhibits Hypoxia-Induced Endothelial Cell Proliferation and Migration

**Figure 4:**
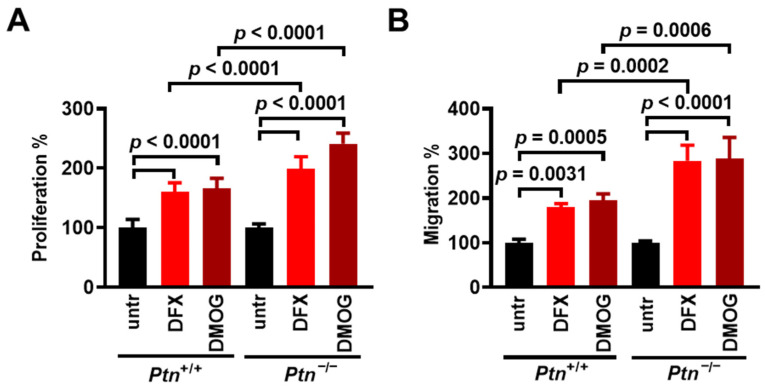
PTN expression inhibits hypoxia-induced endothelial cell proliferation and migration. (**A**) *Ptn*^+/+^ and *Ptn*^−/−^ LMVECs were incubated for 24 h with 0.5 mM of DMOG or 200 µM of DFX and cell numbers were determined by direct counting. Results are expressed as the mean ± standard deviation (n = 3) of the percent (%) number of cells compared to the corresponding untreated cells (set by default as 100%). (**B**) *Ptn*^+/+^ and *Ptn*^−/−^ LMVECs were incubated for 4 h with 0.5 mM of DMOG or 200 µM of DFX, and migration was studied using the transwell assay. Results are expressed as the mean ± standard deviation (n ≥ 3) of the % number of cells compared to the corresponding untreated cells (untr, set by default as 100%).

To investigate whether hypoxia-induced PTN expression has a functional significance in hypoxia-induced endothelial cell proliferation and migration, we used LMVECs isolated from C57BL/6 mice that are a knockout for PTN and studied the effect of chemical hypoxia on their proliferation and migration compared to the wild-type LMVEC. In *Ptn*^+/+^ LMVECs, hypoxia up-regulates the PTN protein levels (Supplementary Figure S3A), similarly to HUVECs. Figure 4A and Figure S3B show that DFX and DMOG induce *Ptn*^−/−^ and *Ptn*^+/+^ LMVEC proliferation, and the effect of chemical hypoxia is significantly higher in *Ptn*^−/−^ compared to *Ptn*^+/+^ LMVECs. Like proliferation, both DFX and DMOG have a substantially greater impact on the migration of *Ptn*^−/−^ compared with *Ptn*^+/+^ LMVECs (Figure 4B and Figure S3C). Besides endothelial cells, in human prostate cancer LNCaP cells that are stably transfected with an antisense sequence and express significantly reduced levels of PTN [22] hypoxia resulted in a significantly higher induction of migration compared with the corresponding cells transfected with the plasmid control vector (Supplementary Figure S4).

### 3.5. Hypoxia Decreases PTN Expression in Glioblastoma Cells That Do Not Express α_ν_β_3_ Integrin

**Figure 5:**
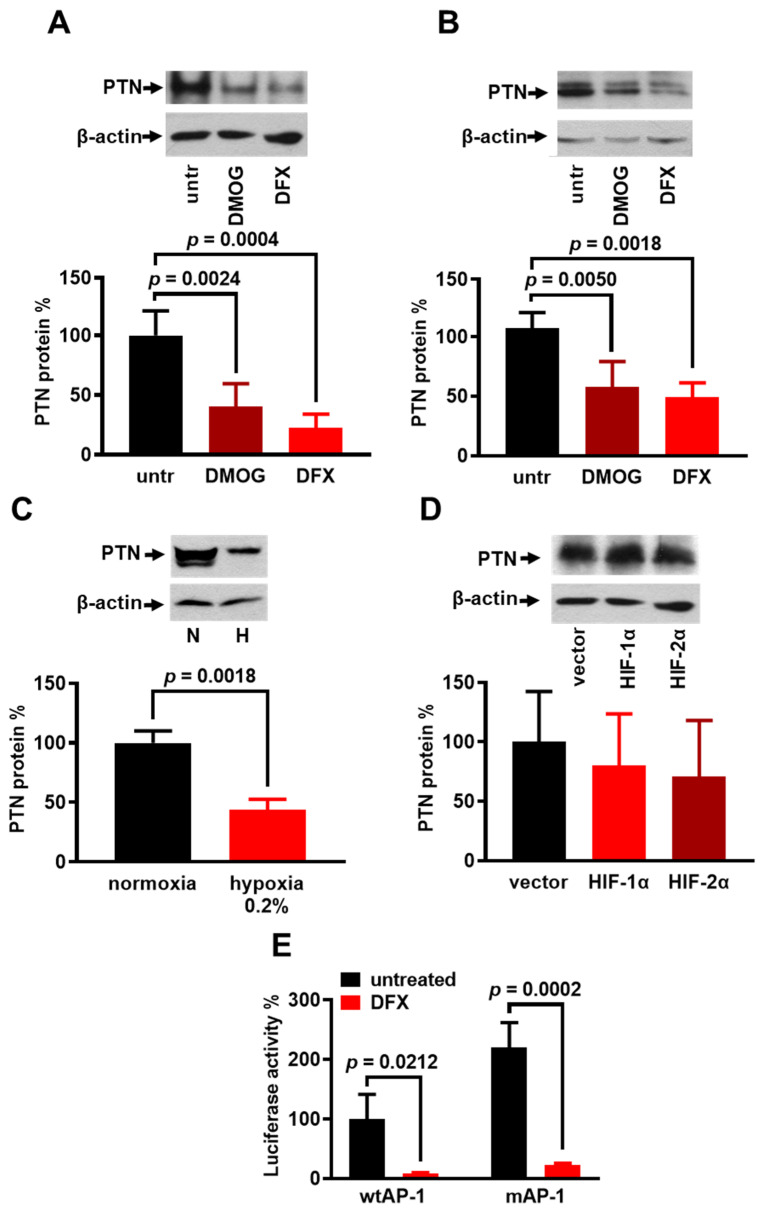
Effect of hypoxia on PTN expression in glioblastoma cells that lack α_ν_β_3_ integrin expression. C6 (**A**) and M059K (**B**) cells were treated with DMOG (0.5 mM) or DFX (200 μM) for 24 h. Cell lysates were analyzed by Western blot for PTN and β-actin, and results are expressed as mean ± SD (n = 4) of percentage change in relative PTN protein levels compared with untreated cells. (**C**) Representative Western blot for PTN and β-actin following 24 h incubation of M059K cells in Anaerocult A bags (hypoxia 0.2%). Results are expressed as mean ± SD (n = 3) of percentage change in PTN protein levels compared with normoxia-treated cells (set by default as 100%). N, normoxia; H, hypoxia. (**D**) C6 cells were transfected with pEGFP-HIF-1α (HIF-1α), pEGFP-HIF-2α (HIF-2α), or pcDNA3.1-GFP vector (vector). Cell lysates were analyzed by Western blot for PTN and β-actin, and results are expressed as mean ± SD (n = 6) of percentage change in PTN protein levels compared with untreated cells. (**E**) M059K cells were transfected with full-length promoter of wild-type human *AP-1* gene (wtAP-1) or full-length promoter of human *AP-1* gene carrying point mutations in both AP-1-like motifs (mutAP-1), fused to luciferase reporter gene. Luciferase activity 24 h after adding DFX in cell culture medium was normalized to total protein content. Results are expressed as mean ± SD (n = 3) of percent change in luciferase activity compared to untreated cells transfected with wtAP-1 construct (set by default as 100%).

We have previously shown that PTN activates cell migration in endothelial and U87MG glioblastoma cells that express α_ν_β_3_ integrin but inhibits cell migration in C6 and M059K cells that do not express α_ν_β_3_ integrin [9,27]. To study whether hypoxia affects the PTN expression in cells that do not express α_ν_β_3_ integrin (Supplementary Figure S5), we incubated C6 and M059K cells with DFX and/or DMOG for 24 h and looked for their effect on PTN expression. Opposite to their stimulatory effect on endothelial cells, DFX and DMOG decreased the PTN protein levels in C6 (Figure 5A) and M059K (Figure 5B) cells. In the latter cells, a hypoxia of 0.2% for 24 h also decreased the PTN protein levels (Figure 5C). The overexpression of HIF-1α and HIF-2α increased VEGFA protein levels as expected (Supplementary Figure S6) but did not affect the PTN protein levels in C6 cells (Figure 5C). AP-1 is not involved in the inhibitory effect of hypoxia, since when we examined the contribution of the AP-1 motifs of the human *ptn* promoter to the increased transcription of the *ptn* gene in DFX-treated M059K cells, we found that chemical hypoxia decreased the reporter activity of both the wild-type (wtAP-1) and the AP-1 double mutant *ptn* (mutAP-1) promoter (Figure 5D).

### 3.6. The Interplay Between Hypoxia and PTPRZ1

**Figure 6:**
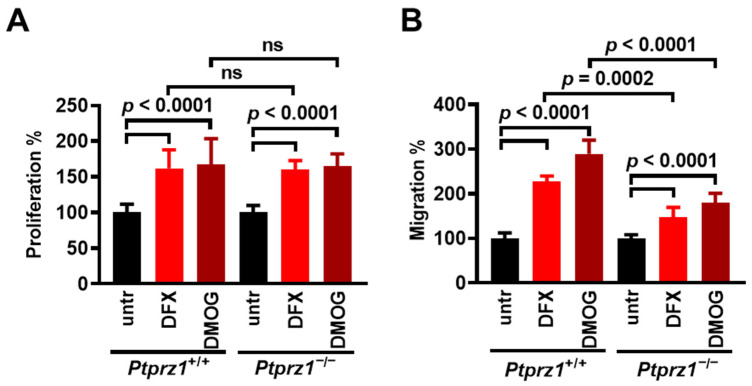
The effect of PTPRZ1 expression on the hypoxia-induced endothelial cell proliferation and migration. (**A**) *Ptprz1*^+/+^ and *Ptprz1*^−/−^ LMVECs were incubated for 24 h with 200 µM of DFX or 0.5 mM of DMOG and cell numbers were determined by direct counting. Results are expressed as the mean ± standard deviation (n = 3) of the % number of cells compared to the corresponding untreated cells (set by default as 100%). ns, non-significant. (**B**) *Ptprz1*^+/+^ and *Ptprz1*^−/−^ LMVECs were incubated for 4 h with DFX or DMOG and migration was studied using the transwell assay. Results are expressed as the mean ± standard deviation (n ≥ 3) of the % number of cells compared to the corresponding untreated cells (set by default as 100%).

Since PTN largely acts through PTPRZ1, which both HIF-1α [20] and HIF-2α [18,19] up-regulate, we studied the effect of chemical hypoxia on the proliferation and migration of LMVECs derived from *Ptprz1*^+/+^ and *Ptprz1*^−/−^ mice. DFX and DMOG significantly enhanced the proliferation (A) and migration (B) of both *Ptprz1*^+/+^ and *Ptprz1*^−/−^ LMVECs. The effect of DFX and DMOG on LMVEC proliferation was similar, independent of the expression of PTPRZ1. However, their impact on *Ptprz1*^−/−^ LMVEC migration was significantly smaller than that on *Ptprz1*^+/+^ LMVECs (Figure 6 and Figure S7). Likewise, in human prostate cancer LNCaP and rat glioma C6 cells, the downregulation of the PTPRZ1 expression by siRNA resulted in a smaller increase in the cell migration induced by hypoxia or DMOG, respectively (Supplementary Figure S8A,B). In both LNCaP and C6 cells, the downregulation of PTPRZ1 significantly enhanced cell migration (Supplementary Figure S8C,D), like what we have observed in *Ptprz1*^−/−^ LMVECs (Supplementary Figure S7 and [12]).

We also studied the effect of chemical hypoxia on the expression of PTPRZ1 in HUVECs and C6 cells. As shown in Supplementary Figure S9, hypoxia or HIF-1α or HIF-2α overexpression increased the protein levels of PTPRZ1 in both types of cells.

## 4. Discussion

In this work, we investigated how hypoxia regulates the PTN expression in endothelial cells and the role of PTN in hypoxia-induced endothelial cell activation. Our data show that hypoxia, including chemical hypoxia, enhances the PTN expression in endothelial cells in line with a previous observation that the PTN expression is elevated in endothelial cells and macrophages in response to acute ischemic brain injuries [17]. PTN shares a 98% homology with midkine, which is up-regulated by hypoxia in pulmonary epithelial cells in a HIF-1α-dependent manner through transcriptional regulation via the proximal HRE in the midkine promoter [30]. The *ptn* gene promoter does not seem to have HREs, based on software programs that search for transcription factor binding sites, such as TFBIND (http://tfbind.hgc.jp/, accessed on 13 December 2024) [31] and the Transcription Factor Target Gene Database [32]; however, we cannot exclude that HREs exist in the 3 region of the *ptn* gene, as is the case, e.g., for the erythropoietin gene [33]. On the other hand, a previous analysis showed that most genes affected by hypoxia lack a HIF-binding site in their promoters [2]. In our study, although the siRNA data suggest that the up-regulation of PTN by hypoxia may be HIF-1α- and HIF-2α-independent, the ODNs for HIFs inhibit the PTN up-regulation by low oxygen levels. One explanation may be that the specific inhibition of the individual HIFs does not inhibit the PTN expression because the other HIF isoform may still be sufficient to activate it. This is supported by our data showing that the overexpression of HIF-1α or HIF-2α in U87MG cells up-regulates the PTN expression. The hypoxia-induced PTN expression in endothelial cells is also AP-1-dependent, in line with previous studies that have shown the interaction of HIFs with members of the AP-1 family of transcription factors to regulate gene transcription under hypoxic conditions [4,34]. Both HIFs and AP-1 are activated by deferoxamine [35], while DMOG stabilizes and thus up-regulates HIFs [1], but it is not known whether it also activates AP-1. The *ptn* gene promoter contains two AP-1 HREs that are required for the up-regulation of PTN by hydrogen peroxide, nitric oxide, and fibroblast growth factor 2 [7,36,37] and, based on the data of the present study, for the up-regulation of *ptn* gene transcription by hypoxia in endothelial and cancer cells that express α_ν_β_3_ integrin.

The enhanced chemical hypoxia-induced proliferation and migration observed in *Ptn*^−/−^ compared with *Ptn*^+/+^ LMVECs agrees with the notion that PTN may act as an endogenous brake for the stimulatory effect of hypoxia in endothelial cells, a hypothesis verified in prostate cancer LNCaP cells with a down-regulated PTN expression. Interestingly, hypoxia inhibits the PTN expression in cells that do not express α_ν_β_3_ integrin. This aligns with numerous studies showing that the gene regulation by hypoxia is cell context-dependent [38] and with our previous observation that the regulation of the PTN expression by VEGFA differs between cells based on the expression of α_ν_β_3_ integrin [28]. Although it is unclear how the presence of α_ν_β_3_ integrin affects how hypoxia impacts PTN expression, the resulting decreased or increased PTN levels align with the stimulatory effect of short-term hypoxia on cell migration. We have previously shown that in cells that do not express α_ν_β_3_ integrin, such as C6 cells, exogenous PTN inhibits cell migration and down-regulation of the endogenous PTN expression significantly enhances it [9,39]. In contrast, in cells that express α_ν_β_3_ integrin, exogenous or endogenous PTN stimulates cell migration [9,21,22].

Although hypoxia differentially affects the PTN expression in different types of cells, it enhances the PTPRZ1 protein levels in all cells studied, in line with previous studies showing that the *Ptprz1* gene contains HREs and its expression is directly up-regulated by both HIF-1α [20] and HIF-2α [18,19]. To investigate the hypothesis that PTPRZ1 may have a functional role in the effects of hypoxia on endothelial cells, we used endothelial cells derived from *Ptprz1*^−/−^ mice or LNCaP or C6 cells following the downregulation of PTPRZ1. The impact of hypoxia on endothelial cell proliferation seems to be independent of the expression of PTPRZ1, aligning with the notion that PTPRZ1 mostly affects cell migration [40]. However, the effect of hypoxia was significantly smaller in the migration of *Ptprz1*^−/−^ LMVECs or in LNCaP and C6 cells, in which the PTPRZ1 expression was down-regulated. This might be explained by the observation that when the expression of PTPRZ1 is decreased, HIF-1α protein levels are increased under normoxic conditions [41] and cell migration is enhanced (this study and [12]). Therefore, the smaller impact of hypoxia on the migration of cells with a decreased PTPRZ1 expression may be attributed to the (already) enhanced basal HIF-1α levels and migration of these cells under normoxic conditions. Altogether, these data suggest that since PTPRZ1 negatively impacts cell migration, the enhancement of the PTPRZ1 expression by hypoxia may act as a negative regulator of HIF activation, restricting the HIF-dependent effects of hypoxia on cell migration.

Our data show that the impact of PTN and PTPRZ1 in the hypoxia-induced cell migration are differential and do not follow the mirror effect expected for a ligand and its receptor. This is not a surprise and complies with the suggestion that PTPRZ1 is a tyrosine phosphatase receptor that, at least in some cases, is inactivated upon PTN binding [11,12]. Therefore, the loss of PTPRZ1 would mimic the effect of PTN, which is evidenced by the observations that *Ptprz1*^−/−^ LMVECs have enhanced migration compared with *Ptprz1*^+/+^ LMVECs and do not respond further to PTN or VEGFA stimulation [12]. On the other hand, the unstimulated *Ptn*^−/−^ LMVECs have a slightly decreased proliferation and migration compared to their corresponding wild-type cells and have an enhanced response to VEGFA [21], which is in line with our previous observations that although PTN has a modest stimulatory effect on endothelial cell migration by itself, it restricts the stimulatory effect of VEGFA on VEGFR2 activation [10] and endothelial cell proliferation [42] and migration [14,28,42]. This may also explain why in *Ptn*^−/−^ LMVECs, hypoxia has an enhanced stimulatory effect compared to the corresponding PTN-expressing LMVECs. It is known that PTN acts through numerous receptors besides PTPRZ1 [11], and PTPRZ1 acts as a receptor that regulates the effects of other cytokines, growth factors, and cell adhesion molecules [13], further supporting that we should not expect a mirror effect.

## 5. Conclusions

**Figure 7:**
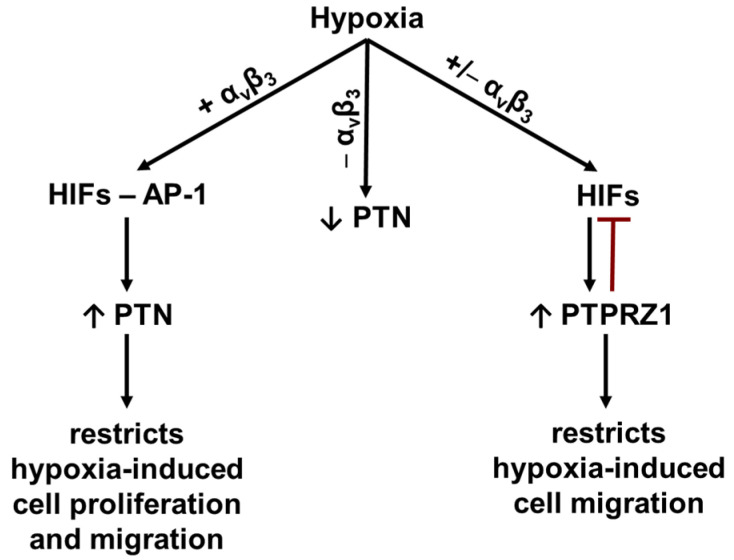
A schematic representation of the effect of hypoxia on the PTN and PTPRZ1 expression in endothelial cells. Hypoxia activates HIFs and AP-1 to up-regulate the PTN expression in α_ν_β_3_ integrin-expressing cells to restrict the stimulatory effect of hypoxia on cell proliferation and migration. In cells that do not express α_v_β_3_ integrin, hypoxia downregulates the PTN expression in a HIF- and AP-1-independent manner. In all cases, hypoxia upregulates the expression of PTPRZ1, which is known to negatively regulate (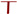) HIF1α [41] and restricts hypoxia-induced cell migration.

Our data suggest that hypoxia regulates the PTN expression in a cell (α_ν_β_3_ integrin)-dependent manner and up-regulates the PTPRZ1 expression in a HIF-dependent manner. However, through different mechanisms, both PTN and PTPRZ1 seem to restrict the stimulatory effect of hypoxia on cell migration, favoring the notion that they act as endogenous brakes for excessive cell migration (Figure 7).

## Data Availability

All data supporting this study’s findings are available from the corresponding author upon request.

## Supplementary Materials

The following supporting information can be downloaded at https://www.mdpi.com/article/10.3390/cancers17091516/s1: Table S1. Sequences of the single-stranded ODNs used in the present study; Figure S1A. Effect of different concentrations of DMOG and DFX (200 μM) on PTN protein levels in HUVECs; Figure S1B. Effect of DFX (200 μM) on PTN protein levels in U87MG cells; Figure S2A. Efficiency of cell transfection with siRNAs for HIF-1α or HIF-2α. HIF-1α or HIF-2α mRNA levels 18 h after transfection with the corresponding siRNAs (siHIF-1α or siHIF-2α); Figure S2B. Overexpression of HIF-1α and HIF-2α in U87MG cells; Figure S3. Effect of endogenous PTN expression on chemical hypoxia-induced proliferation and migration of LMVECs; Figure S4. Effect of endogenous PTN expression in hypoxia-induced migration of prostate cancer LNCaP cells; Figure S5. Expression of the α_ν_β_3_ integrin in the cells used in the present study; Figure S6. Overexpression of HIF-1α and HIF-2α in C6 cells; Figure S7. Effect of endogenous PTPRZ1 expression in chemical hypoxia-induced proliferation and migration of LMVECs; Figure S8. Effect of PTPRZ1 expression on the hypoxia-induced cell migration; Figure S9. Hypoxia enhances the expression of PTPRZ1 in all types of cells.

## Abbreviations

The following abbreviations are used in this manuscript:

HIF	hypoxia-inducible transcription factor
PTN	pleiotrophin
PTPRZ1	protein tyrosine phosphatase receptor zeta 1
VEGFA_165_	vascular endothelial growth factor A 165
