# Supplementary material for: Regulation of Pleiotrophin and PTPRZ1 Expression by Hypoxia to Restrict Hypoxia-Induced Cell Migration
Source: Cancers (Basel). 2025 Apr 30;17(9):1516. doi: 10.3390/cancers17091516 (PMC12070880; doi:10.3390/cancers17091516)
Supplement: Supplementary file 1 [file cancers-17-01516-s001.zip › cancers-3571093-supplementary.pdf]

Regulation of Pleiotrophin and PTPRZ1 Expression by Hypoxia to Restrict Hypoxia-Induced Cell Migration

Evangelia Poimenidi[1,2,#], Eirini Droggiti[1], Katerina Karavasili[1], Dimitra Kotsirilou[1,2], Eleni Mourkogianni[1], Pieter Koolwijk[2], Evangelia Papadimitriou[1,*]

[1]Laboratory of Molecular Pharmacology, Department of Pharmacy, University of Patras, Patras 26504, Greece; [2]Department of Physiology, Amsterdam UMC, Amsterdam, The Netherlands

[#]Current address: Anaesthesia and Intensive Care Medicine, Leicester Royal Infirmary, University Hospitals of Leicester, Leicester, UK.

Table S1. Sequences of the single-stranded ODNs used in the present study [7,29]. Bold and underlined letters denote the consensus and mutated AP-1 or HIF binding sites.

AP-1	**5′-CGCTTGA<u>TGACTCA</u>GCCGGAA-3′**
mutant AP-1	**5′-CGCTTGA<u>TTACTTA</u>GCCGGAA-3′**
HIF	**5′-GCCCT<u>ACGTG</u>CTGTCTCA-3′**
mutant HIF	**5′-GCCCT<u>TACAA</u>CTGTCTCA -3′**

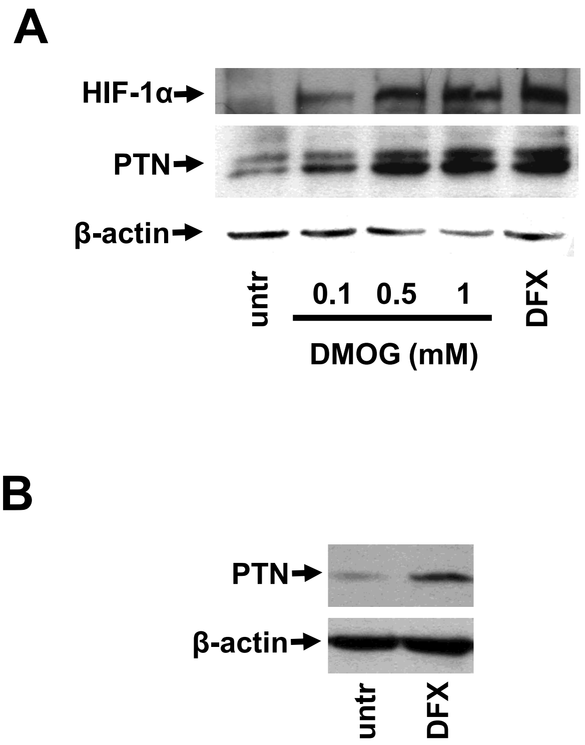

Figure S1. (A) Effect of different concentrations of DMOG and DFX (200 µM) on PTN protein levels in HUVECs. Representative Western blot for HIF-1α, PTN, and β-actin following a 24-hour HUVECs treatment with different concentrations of DMOG or 200 µM DFX. **(B) Effect of DFX (200 µM) on PTN protein levels in U87MG cells.** Representative Western blot for PTN and β-actin following a 24-hour treatment of U87MG cells with DFX.

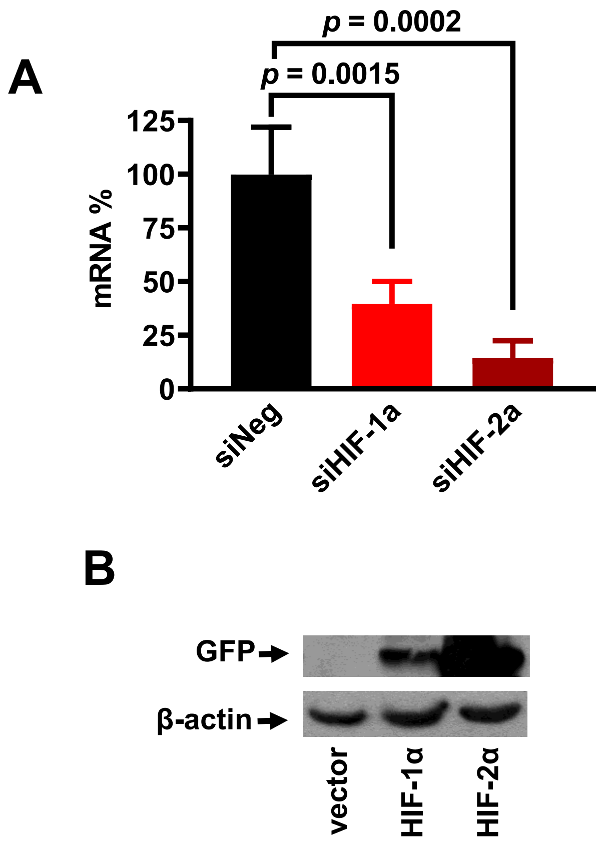

Figure S2. (A) Efficiency of cell transfection with siRNAs for HIF-1α or HIF-2α. HIF-1α or HIF-2α mRNA levels 18 h after transfection with the corresponding siRNAs (siHIF-1α or siHIF-2α). siNeg corresponds to cells treated with a negative control siRNA sequence. Results are expressed as mean ± SD (n=3) of the percent change of HIF mRNA levels compared to the siNeg-treated cells. **(B) Overexpression of HIF-1α and HIF-2α in U87MG cells.** Representative Western blot for GFP and β-actin following transfection of U87MG cells with the GFP-HIF plasmids.

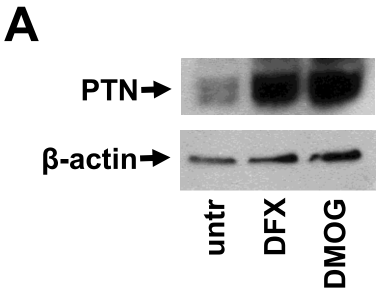

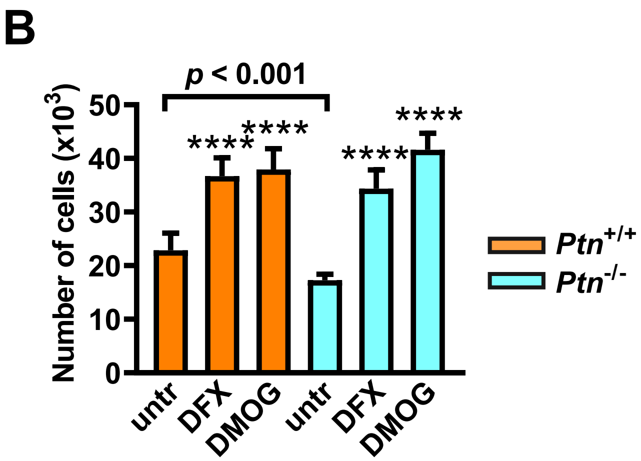

	Ptn$^{+/+}$	*Ptn*$^{-/-}$
untreated	22.83 ± 3.21 (100%)	17.25 ± 1.13 (100%)
DFX	36.67 ± 3.44 (161%)	34.33 ± 3.52 (199%)
DMOG	37.92 ± 3.89 (166%)	41.58 ± 3.08 (241%)

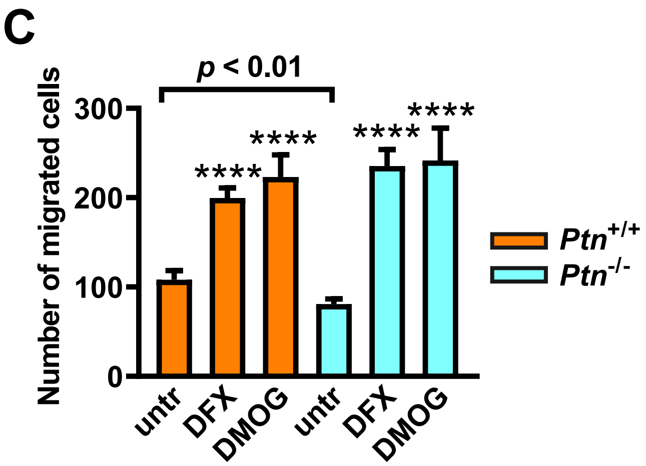

	Ptn$^{+/+}$	*Ptn*$^{-/-}$
untreated	108.1 ± 10.33 (100%)	80.9 ± 5.70 (100%)
DFX	199.7 ± 11.15 (185%)	235.3 ± 18.58 (291%)
DMOG	223.0 ± 24.76 (206%)	241.7 ± 36.17 (299%)

Figure S3. Effect of endogenous PTN expression on chemical hypoxia-induced proliferation and migration of LMVECs. (A) Representative Western blot for PTN and β-actin following a 24 h incubation of *Ptn*$^{+/+}$ LMVECs with DFX (200 µM) or DMOG (0.5 mM). **(B)** *Ptn*$^{+/+}$ and *Ptn*$^{-/-}$ LMVECs were incubated for 24 h with 0.5 mM DMOG or 200 µM DFX and cell numbers were determined by direct counting. Results are expressed as mean ± standard deviation (n=3) of the number of cells. **(C)** *Ptn*$^{+/+}$ and *Ptn*$^{-/-}$ LMVECs were incubated for 4 h with DMOG or DFX and migration was studied using the transwell assay. Results are expressed as mean ± standard deviation (n≥3) of the number of cells that migrated through the filter. In both B and C, asterisks denote statistical significance compared with the corresponding untreated (untr) LMVECs. **** $p < 0.0001$. Parentheses in the tables show the percent change in the number of cells compared with the corresponding untreated cells.

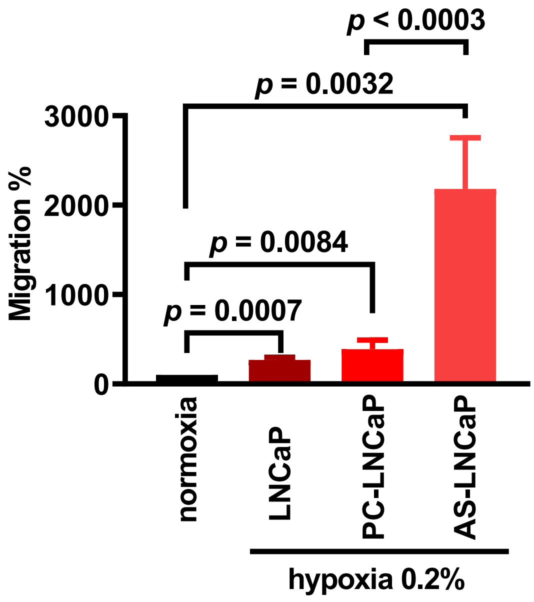

Figure S4. Effect of endogenous PTN expression in hypoxia-induced migration of prostate cancer LNCaP cells. Results are expressed as mean ± S.D. of the % number of migrated cells compared to the corresponding normoxia-treated cells. LNCaP, non-transfected LNCaP cells; PC-LNCaP, LNCaP cells transfected with the plasmid containing only the neomycin resistance gene; AS-LNCaP, LNCaP cells transfected with the plasmid containing antisense PTN.

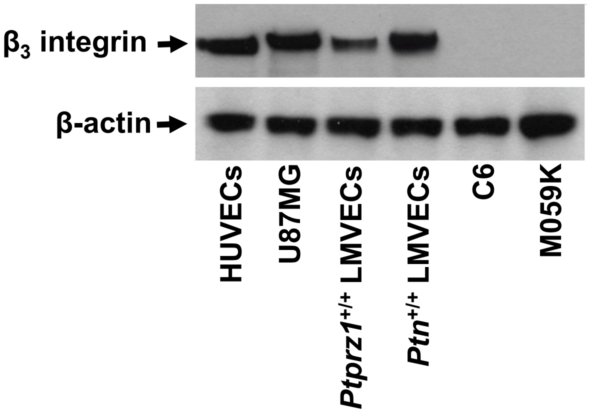

Figure S5. Expression of the $\alpha_v\beta_3$ integrin in the cells used in the present study. Representative Western blot for β_3 integrin and β-actin in cell lysates.

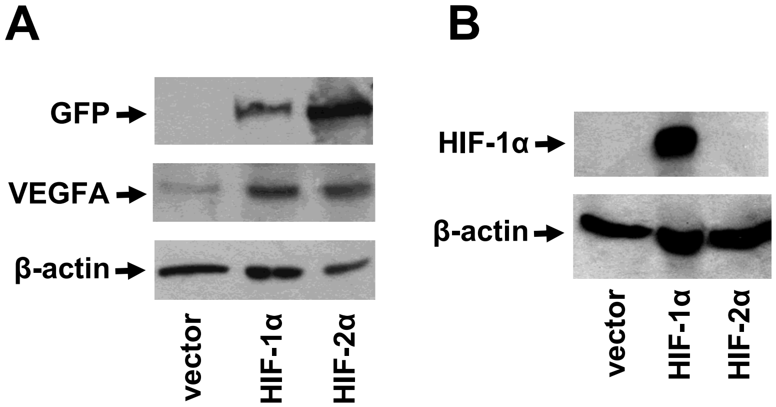

Figure S6. Overexpression of HIF-1α and HIF-2α in C6 cells. (A) Representative Western blot for GFP, VEGFA, and β-actin following transfection of C6 cells with the GFP-HIF plasmids. **(B)** Representative Western blot for HIF-1α and β-actin following transfection of C6 cells with the GFP-HIF plasmids.

A

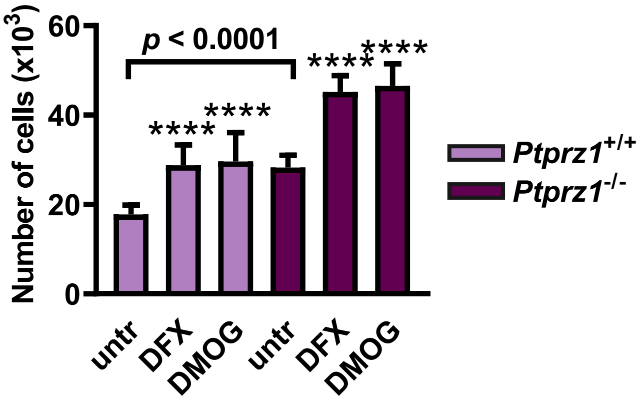

	Ptprz1$^{+/+}$	*Ptprz1*$^{-/-}$
untreated	17.75 ± 2.10 (100%)	28.25 ± 2.77 (100%)
DFX	28.75 ± 4.57 (162%)	45.17 ± 3.63 (160%)
DMOG	29.63 ± 6.47 (167%)	46.54 ± 4.95 (165%)

B

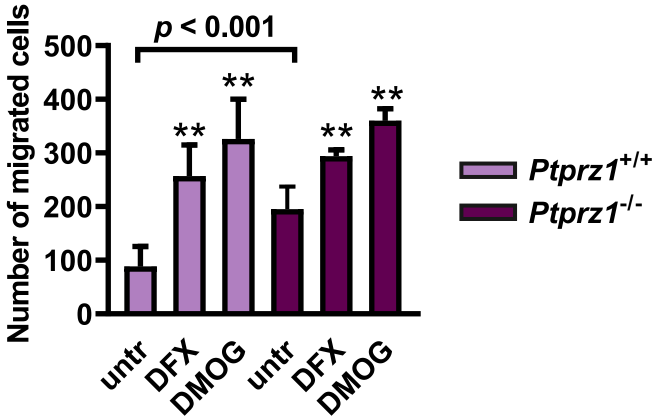

	Ptprz1$^{+/+}$	*Ptprz1*$^{-/-}$
untreated	88.4 ± 37.12 (100%)	195.2 ± 41.90 (100%)
DFX	257.0 ± 57.63 (291%)	293.8 ± 11.90 (150%)
DMOG	326.0 ± 74.09 (369%)	360.0 ± 22.45 (185%)

Figure S7. Effect of endogenous PTPRZ1 expression in chemical hypoxia-induced proliferation and migration of LMVECs. (A) *Ptprz1*$^{+/+}$ and *Ptprz1*$^{-/-}$ LMVECs were incubated for 24 h with 0.5 mM DMOG or 200 μM DFX and cell numbers were determined by direct counting. Results are expressed as mean ± standard deviation (n=3) of the number of cells. **(B)** *Ptprz1*$^{+/+}$ and *Ptprz1*$^{-/-}$ LMVECs were incubated for 4 h with DMOG or DFX and migration was studied using the transwell assay. Results are expressed as mean ± standard deviation (n≥3) of the number of cells that migrated through the filter. In both cases, asterisks denote statistical significance compared with the corresponding untreated (untr) LMVECs. ** $p < 0.01$, **** $p < 0.0001$. Parentheses in the tables show the percent change in the number of cells compared with the corresponding untreated cells.

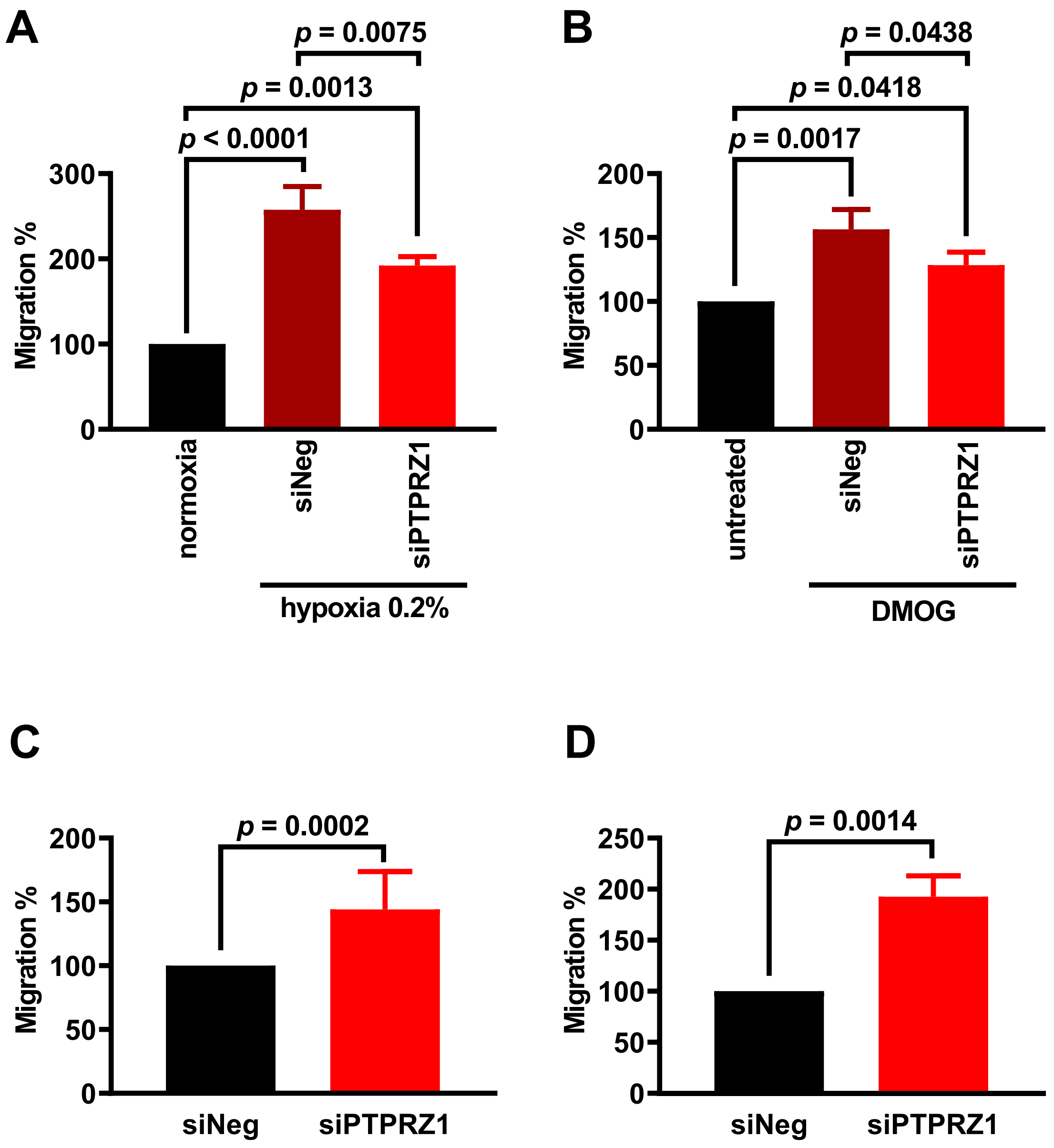

Figure S8. Effect of PTPRZ1 expression on the hypoxia-induced cell migration. (A and C) Human prostate cancer LNCaP cells were incubated for 4 h in normoxic conditions or Anaerocult A bags (hypoxia 0.2%). **(B and D)** Rat glioma C6 cells were treated with DMOG (0.5 mM), and migration was studied using the transwell assay. Results in A and B are expressed as the mean ± standard deviation (n=3) of the % number of migrated cells compared with the corresponding normoxia-treated or untreated cells (set by default as 100%). Results in C and D are expressed as the mean ± standard deviation (n=10 in C6 cells and n=3 in LNCaP cells) of the % number of migrated cells compared with the corresponding siNeg cells (set by default as 100%). siNeg, cells treated with a negative control siRNA sequence; siPTPRZ1, cells following down-regulation of PTPRZ1 by siRNA.

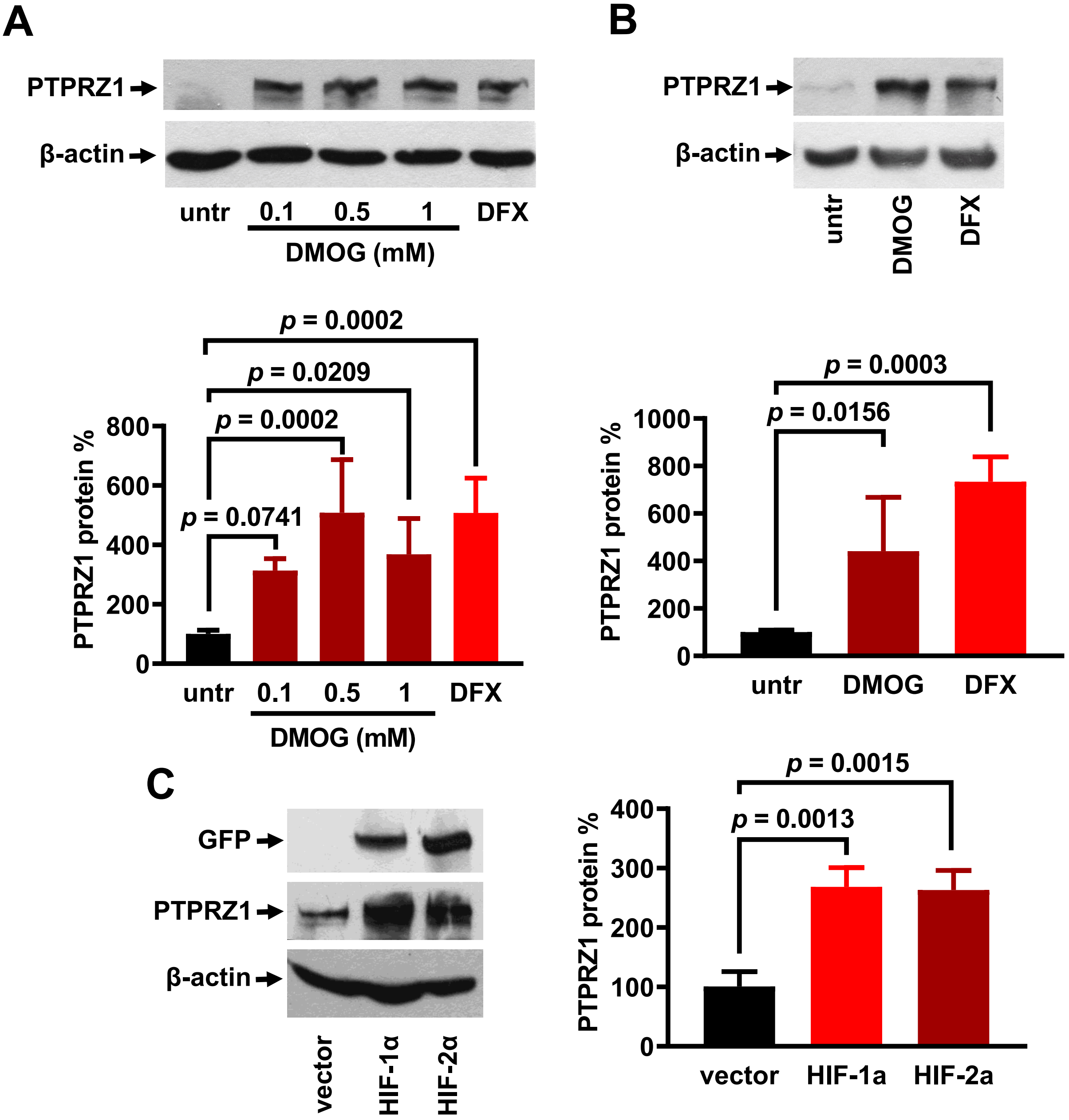

Figure S9. Hypoxia enhances the expression of PTPRZ1 in all types of cells. (A) Representative Western blot for PTPRZ1 and β-actin following a 24-hour treatment of HUVEC with different concentrations of DMOG or DFX (200 μM) **(B)**. Representative Western blot for PTPRZ1 and β-actin following a 24-hour treatment of C6 cells with DMOG (0.5 mM) or DFX (200 μM). **(C)** Representative Western blot for PTPRZ1 and β-actin following transfection of C6 cells with pEGFP-HIF-1α (HIF-1α), pEGFP-HIF-2α (HIF-2α) or the pcDNA3.1-GFP vector (vector). In all panels, PTPRZ1 protein amounts were quantified by densitometric analysis of the corresponding band in each lane. Results are expressed as mean ± SD (n=6) of the percentage change of the PTPRZ1 protein levels compared with the untreated (untr) or vector-treated cells.
